# Supplementary material for: A Process Evaluation of the UK Randomised Trial Evaluating ‘iSupport’, an Online e-Health Intervention for Adult Carers of People Living with Dementia
Source: Behav Sci (Basel). 2025 Aug 15;15(8):1107. doi: 10.3390/bs15081107 (PMC12382822; doi:10.3390/bs15081107)
Supplement: Supplementary file 1 [file behavsci-15-01107-s001.zip › Supplementary File S1.pdf]

SUPPLEMENTARY FILE S1 – iSupport LOGIC MODEL

Inputs	Outputs		Outcomes - Impact		
	Activities	***Participation***	***Short (0-2 yrs.)***	***Medium (3-4 yrs.)***	***Long (5+ yrs.)***
iSupport online intervention<br><br>**Contextual factors:**<br><br>▪ There is a need to investigate the benefits of eHealth interventions (e.g., *iSupport*) for dementia carers<br>▪ Socioeconomic variables (residence, age, SES)<br>▪ Individual resources and capabilities (literacy, ICT experience, community involvement)<br>▪ Individual motivation to engage in *iSupport*<br>▪ Health and care providers - institutional culture (willingness to accept ehealth interventions)	*WHAT WE DO*<br><br>▪ *iSupport* instruction video<br>▪ *iSupport* (UK version including 5 modules)<br>▪ PPI sessions (to inform trial delivery and implementation)<br>▪ Collaborator recruitment activities (e.g., Carers Trust)<br>▪ *iSupport* e-coach<br>▪ Stakeholder events	*WHO WE REACH?*<br><br>▪ 386 participants (of diverse backgrounds and demographics)<br>▪ Third sector collaborators (Alzheimer's Scotland; Carers Trust Wales)<br>▪ Dementia & Ageing researchers across the UK and the world (liaison with the WHO and other research groups doing similar work with *iSupport*)<br>▪ PPI groups<br>▪ NHS clinicians<br>▪ 300 people attending stakeholder/public events	**New knowledge**<br>▪ On the effectiveness of *iSupport*<br>▪ About dementia care for inter/national audiences, including the public<br>▪ On the barriers and facilitators to accessing online training for dementia carers<br>▪ Recommendations to enhance future uptake of *iSupport*<br><br>**Behaviour**<br>▪ The WHO uses the research to inform international implementation of *iSupport*<br>▪ Members of the public confident to be part of research project<br>▪ Dementia carers in the UK feel better able to provide care<br>▪ Dementia carers in the UK have better mental health<br>▪ Health and care providers feel confident to recommend *iSupport* (uptake and acceptance)	**Knowledge**<br>▪ Improved policy and practice around delivering support to dementia carers<br>▪ Increased awareness and uptake of *iSupport* in the world<br><br>**Behaviour**<br>▪ Dementia carers worldwide feel better able to provide care<br>▪ Dementia carers in the UK have good mental health<br>▪ NICE recommend iSupport in dementia guidelines<br>▪ Public Health Wales recommend iSupport in Matrics Cymru<br>▪ Members of the public become research collaborators<br><br>**Opportunity**<br>The research makes a difference to:<br>▪ The global delivery and evidence for implementation of *iSupport* by the WHO and provides a template for those wishing to evaluate *iSupport* in different countries<br>▪ The development of online support interventions for cares and people affected by other chronic health conditions (e.g., Stroke, MS)	**Economic**<br>▪ Reduction in care home admissions<br>▪ Reduced health and social care costs<br>▪ Carers in better health and able to remain in employment<br>▪ High quality research leading to income for UK institutions in next REF<br><br>**Behaviour**<br>Dementia carers are better supported by health and care professionals<br><br>**Opportunity**<br>▪ Sustainability - *iSupport* embedded and recommended as part of care package in UK<br>▪ iSupport (inc. Welsh version) freely available in the UK<br>▪ Other online support packages for people affected by other chronic conditions in being tested

Source: Hage et al. (2013) and McLaughlin & Jordan (1999)
