# Supplementary material for: A Process Evaluation of the UK Randomised Trial Evaluating ‘iSupport’, an Online e-Health Intervention for Adult Carers of People Living with Dementia
Source: Behav Sci (Basel). 2025 Aug 15;15(8):1107. doi: 10.3390/bs15081107 (PMC12382822; doi:10.3390/bs15081107)
Supplement: Supplementary file 1 [file behavsci-15-01107-s001.zip › Supplementary File S2.pdf]

iSupport Online Evaluation v2

Page 1: Please tell us about your experiences of using iSupport

1. **Please let us know what you think about the following statements:**

	Strongly disagree	Disagree	Neither agree or disagree	Agree	Strongly agree
I think that I would like to use iSupport frequently	☐	☐	☐	☐	☐
I found iSupport unnecessarily complex	☐	☐	☐	☐	☐
I thought iSupport was easy to use	☐	☐	☐	☐	☐
I think that I would need the support of a technical person to be able to use iSupport	☐	☐	☐	☐	☐
I found the various functions in iSupport were well integrated	☐	☐	☐	☐	☐

I thought there was too much inconsistency in iSupport	☐	☐	☐	☐	☐
I would imagine that most people would learn to use iSupport very quickly	☐	☐	☐	☐	☐
I found iSupport very cumbersome ('bulky') to use	☐	☐	☐	☐	☐
I felt very confident in using iSupport	☐	☐	☐	☐	☐
I needed to learn a lot of things before I could get going with iSupport	☐	☐	☐	☐	☐

Page 2

2. **Please think about any challenges that you might have encountered when using iSupport and rate their importance (1 being not important at all and 5 extremely important):** ✱ *Required*

	1 (Not important)	2	3	4	5 (Extremely important)
Amount of time needed to use iSupport	☐	☐	☐	☐	☐
Slow internet access	☐	☐	☐	☐	☐
Use and complexity of the virtual platform	☐	☐	☐	☐	☐
Problems with my equipment (e.g. computer)	☐	☐	☐	☐	☐
Lack of confidence/experience in using technology	☐	☐	☐	☐	☐

3. Are there any other challenges of using iSupport that you would like to mention? Please specify:

4. **Please think about advantages in using iSupport and rate their importance (1 being not important at all and 5 extremely important):** ✱ *Required*

	1 (Not important)	2	3	4	5 (Extremely important)
Autonomy and self-paced programme	☐	☐	☐	☐	☐
Access to diverse sources of information	☐	☐	☐	☐	☐
Ability to choose the most relevant modules/sessions/activities	☐	☐	☐	☐	☐
Ability to use iSupport from my own home	☐	☐	☐	☐	☐

5. Are there any other advantages of using iSupport that you would like to mention? Please specify:

6. **Do you think that using iSupport has improved any of the following? Please give a rating** ✱ *Required*

	1 (Not at all)	2	3	4	5 (Very much)
Anxiety	☐	☐	☐	☐	☐
Depression	☐	☐	☐	☐	☐
Stress levels	☐	☐	☐	☐	☐
Feelings of burden	☐	☐	☐	☐	☐
Sleep	☐	☐	☐	☐	☐

Relationship with the person that you care for	☐	☐	☐	☐	☐
Your confidence a a carer	☐	☐	☐	☐	☐

7. **Did you complete all five modules of iSupport?** ✱ *Required*

- ○ No
- ○ Yes

8. If 'no' please tell us why:

- ○ Some modules were not relevant to me
- ○ I didn't have enough time
- ○ Other

8.a. If you selected 'other', please specify:

9. **Please think about how relevant the iSupport modules are to your role as a carer:** ✱ *Required*

	1 (Not relevant at all)	2	3	4	5 (Extremely relevant)	I didn't complete this module
Module 1: Introduction to dementia	☐	☐	☐	☐	☐	☐
Module 2: Being a carer	☐	☐	☐	☐	☐	☐
Module 3: Caring for me	☐	☐	☐	☐	☐	☐
Module 4: Providing everyday care	☐	☐	☐	☐	☐	☐
Module 5: Dealing with behaviour changes	☐	☐	☐	☐	☐	☐

10. **How often did you access iSupport?** ✱ *Required*

- ○ Once a day
- ○ Once a week
- ○ Twice a week
- ○ As and when needed
- ○ Other

10.a. If you selected 'other', please specify:

11. If you requested any technology support/help from a member of our staff regarding using the iSupport website, was the help you received appropriate and effective? ✱ *Required*

- ○ Strongly disagree
- ○ Disagree
- ○ Neither agree or disagree
- ○ Agree
- ○ Strongly agree
- ○ I did not need to ask for help

12. **How did you access the iSupport programme? Please think about how much you used any of the following devices:** ✱ *Required*

	1 Never	2	3	4	5 Always
Mobile phone	☐	☐	☐	☐	☐
Tablet	☐	☐	☐	☐	☐
Laptop computer	☐	☐	☐	☐	☐
Desktop computer	☐	☐	☐	☐	☐

13. **From where did you access the course? Please think about how often you accessed the course from each of the following settings:** ✱ *Required*

	1 Never	2	3	4	5 Always
Home	☐	☐	☐	☐	☐
Workplace	☐	☐	☐	☐	☐
Public transportation	☐	☐	☐	☐	☐

Public spaces (e.g., internet cafe, public library)	☐	☐	☐	☐	☐

13.a. If you accessed iSupport from any other setting, please specify:

Page 3

14. Please let us know what you think about the following statements:

Please don't select more than 1 answer(s) per row.

Please select exactly 16 answer(s).

Please don't select more than 16 answer(s) in any single column.

	Strongly disagree	Disagree	Neither agree or disagree	Agree	Strongly agree
I can see how iSupport is different from other educational tools that talk about dementia	☐	☐	☐	☐	☐
I was able to use iSupport without any help	☐	☐	☐	☐	☐
I consider that most carers would be able to use iSupport without help	☐	☐	☐	☐	☐
The language used in iSupport was easy to understand	☐	☐	☐	☐	☐
The examples used in iSupport to explain things were clear	☐	☐	☐	☐	☐
iSupport feels very familiar	☐	☐	☐	☐	☐

I understand how using iSupport will change the way I see my role as a carer	☐	☐	☐	☐	☐
I liked using iSupport to learn about dementia	☐	☐	☐	☐	☐
I liked using iSupport to learn about my role as a carer	☐	☐	☐	☐	☐
iSupport has taught me something about dementia	☐	☐	☐	☐	☐
iSupport has taught me something about my caring role	☐	☐	☐	☐	☐
I can easily apply the content of iSupport to my daily role as a carer	☐	☐	☐	☐	☐
I can see the potential value of iSupport for my job as a carer	☐	☐	☐	☐	☐
I am happy to recommend iSupport to others (as a tool to help people in a similar situation to mine)	☐	☐	☐	☐	☐
I will continue to use iSupport in the future	☐	☐	☐	☐	☐

I have the right resources (e.g. internet connection) to be able to use iSupport	☐	☐	☐	☐	☐

Page 4: Thank you very much for completing this survey!
