# Supplementary material for: A Process Evaluation of the UK Randomised Trial Evaluating ‘iSupport’, an Online e-Health Intervention for Adult Carers of People Living with Dementia
Source: Behav Sci (Basel). 2025 Aug 15;15(8):1107. doi: 10.3390/bs15081107 (PMC12382822; doi:10.3390/bs15081107)
Supplement: Supplementary file 1 [file behavsci-15-01107-s001.zip › Supplementary File S3.pdf]

Discussing my experiences of using iSupport for Dementia Carers

Example interview schedule

The process evaluation interview schedule will be finalised in collaboration with our partners and PPI group. This example shows the areas for discussion we anticipate we will cover in the final interview schedule.

Introduction:

Many thanks for taking the time to help with this interview.

This interview is being carried out to gather information about the experiences and opinions of participants in the iSupport for Dementia Carers project. If at any time you want to stop, have a break, or if you don't want to answer a question please let me know. Please be assured that everything you say will remain confidential. Extracts of what you say may be included in publications or reports, but your name or any other confidential details will not be linked to it.

Can I ask you to confirm that you are happy to continue, and for the conversation to be recorded? If you are not, I can make notes instead.

1. Can you tell me a bit about your experiences of being a carer for someone living with dementia?

1.1 *How long have you supported someone living with dementia?*

1.2 *In what ways do you support them? What type of caring duties do you have?*

1.3 *How does it impact your day-to-day life?*

1.4 *What support have you received from others? (professionals, other family members, friends) How would you rate it? Has it been useful?*

1.5 *How has COVID-19 impacted your caring role?*

[Acceptability]

2. Before you started, how did you feel about iSupport?

2.1. *Can you tell me a little bit about why you decided to join this study? Where did you hear about it?*

2.2. *Had you heard about iSupport before?*

2.2 *Did you think there is a need for this type of website?*

2.3 *Did you know how iSupport might help you?*

2.4. *Was the information you received from the research team clear as to how to access and use iSupport? Did you feel ready?*

2.5. *What made you be interested in joining the trial? Where did you hear about it?*

3. **How often did you access iSupport?**

3.1. *Did you encounter any problems fitting accessing iSupport into your daily/normal routine?*

3.2. *How much effort did you feel it took to use iSupport?*

3.3. *Would text reminders/emails (or any other form of reminder) influence how often you use? Why?*

3.4. *How did you engage with iSupport? Did you log in several times for a short amount of time, or did you complete it in a few longer sessions? Why?*

3.5. *Did you follow the module order, or did you jump from modules depending on what you were interested in?*

4. **To what extent does iSupport fit in with your value system and with your culture?**
5. **To what extent have you had to give up on something (e.g. doing something you like, time, work) to be able to participate in iSupport?**
6. **How confident were you that you would be able to understand and relate to the content of iSupport?**
7. **How confident were you that you had the technological skills required to participate in iSupport?**
8. **How confident were you that iSupport would achieve its purpose?**
9. **How confident were you that you would be able to use/apply what you learnt from iSupport in your everyday life?**
10. **Do you think iSupport is particularly useful in current COVID times? Why?**

10.1. *Do you think iSupport 'makes more sense' now that we are dealing with a pandemic? Do you think it can help support carers if we are ever again in a similar situation? Why?*

[Usability and experience of use]

11. **What was your overall impression of using iSupport?**
12. **What were the most positive aspects/advantages?**
13. **If any, which were the most negative/challenging aspects?**

13.1. *Did you have any issues with technology?*

13.2. *Did you need to contact the e-coach? How often did you have contact with the e-coach?*

14. Was iSupport easy to access? (including 'read aloud function')

14.1. *Did you have any problems logging on?*

14.2. *Do you think most carers would have been able to follow the logging on steps?*

15. Was iSupport easy to use?

15.1. *To what extent do you consider that most carers would be able to use the program without help?*

15.2. *Did you find any error or problem when using the program? If so, how did you resolve it?*

16. What do you think about the 'look' (visual appearance) of iSupport?

16.1. *Was it appealing to look at?*

16.2. *What do you think about the design and images used? Did you find they fit with your value system and cultural background?*

16.3. *What do you think about how the text was organized?*

17. What is your opinion about the language used?

17.1. *To what extent do you consider the language to be plain and accessible to most carers?*

17.2. *Did you find any negative, offensive, or inadequate term or expression? Did the language used fit with your value system and cultural background?*

18. What are your thoughts about the content of the modules?

18.1. *What do you think about the usefulness or relevance of the modules?*

18.2. *Was there any kind of information that you did not find helpful? Can you think of an example?*

18.3. *Was there any kind of information that was missing? Can you think of an example?*

18.4. *Was there any information that you found unnecessary or inadequate?*

[Mechanisms of change – perceived benefits/expected outcomes]

19. What impact has iSupport had, if any, in your health and wellbeing?

20. What is it about iSupport that has contributed to these changes/impact?

***20.1** What aspects of iSupport, if any, do you think have helped the most in making a difference to the way you deal with your caring role?*

21. What do you feel was the most significant change in the way you deal with your caring role after accessing iSupport?

***21.1.** Have you learnt more about how to deal with your caring role? In what way?*

***21.2.** Have you implemented anything you learnt in iSupport into your day-to-day activities? Can you think of an example?*

***21.3.** In what way, if any has iSupport impacted the person you are caring for and your relationship with him/her?*

22. Would you recommend iSupport to other carers? Why?

***22.1.** Which carers do you think would benefit most from using iSupport?*

To end: I have asked you all the questions I wanted to ask you, is there anything you would like to say or any other issue I haven't mentioned that you would like to discuss?

Thank you very much for your help.
