# Supplementary material for: A Process Evaluation of the UK Randomised Trial Evaluating ‘iSupport’, an Online e-Health Intervention for Adult Carers of People Living with Dementia
Source: Behav Sci (Basel). 2025 Aug 15;15(8):1107. doi: 10.3390/bs15081107 (PMC12382822; doi:10.3390/bs15081107)
Supplement: Supplementary file 1 [file behavsci-15-01107-s001.zip › Supplementary File S4.pdf]

WS2 – Qualitative Interview Data analysis - INITIAL CODING FRAMEWORK

ACCEPTABILITY	PROPOSED INITIAL CODES
**1. Can you tell me a bit about your experiences of being a carer for someone living with dementia?**<br>**1.1** How long have you supported someone living with dementia?<br>**1.2** In what ways do you support them? What type of caring duties do you have?<br>**1.3** How does it impact your day-to-day life?<br>**1.4** What support have you received from others? (professionals, other family members, friends) How would you rate it? Has it been useful?<br>**1.5** How has COVID-19 impacted your caring role?	▪ CARING EXPERIENCES<br>▪ CARING DUTIES<br>▪ IMPACT OF COVID (on caring role)<br>▪ SUPPORT NETWORK - CARER
**2. Before you started, how did you feel about iSupport?**<br>**2.1.** Can you tell me a little bit about why you decided to join this study? Where did you hear about it?<br>**2.2.** Had you heard about iSupport before?<br>**2.2** Did you think there is a need for this type of website?<br>**2.3** Did you know how iSupport might help you?<br>**2.4.** Was the information you received from the research team clear as to how to access and use iSupport? Did you feel ready?<br>**2.5.** What made you be interested in joining the trial? Where did you hear about it?<br><br>**3. How often did you access iSupport?**<br>**3.1.** Did you encounter any problems fitting accessing iSupport into your daily/normal routine?<br>**3.2.** How much effort did you feel it took to use iSupport?<br>**3.3.** Would text reminders/emails (or any other form of reminder) influence how often you use? Why?<br>**3.4.** How did you engage with iSupport? Did you log in several times for a short amount of time, or did you complete it in a few longer sessions? Why?<br>**3.5.** Did you follow the module order, or did you jump from modules depending on what you were interested in?<br>**4. To what extent does iSupport fit in with your value system and with your culture?**<br>**5. To what extent have you had to give up on something (e.g., doing something you like, time, work) to be able to participate in iSupport?**<br>**6. How confident were you that you would be able to understand and relate to the content of iSupport?**<br>**7. How confident were you that you had the technological skills required to participate in iSupport?**<br>**8. How confident were you that iSupport would achieve its purpose?**<br>**9. How confident were you that you would be able to use/apply what you learnt from iSupport in your everyday life?**	▪ MOTIVATION TO JOIN iSUPPORT<br>▪ WHERE/HOW DID YOU HEAR ABOUT ISUPPORT<br>▪ KNOWLEDGE/EXPECTATIONS OF IMPACT OF ISUPPORT – Prior to start using it<br>▪ LEVEL OF CONFIDENCE (Technology) – Prior to start using it<br>▪ LEVEL OF CONFIDENCE (Relatability) – Prior to start<br>▪ IDENTIFIED NEED FOR iSUPPORT<br>▪ READY TO START?<br>▪ FREQUENCY OF LOGGING ON<br>▪ PATTERN OF LOGGING ON and USE<br>▪ REMEMBERING TO LOG ON (issues around)<br>▪ Burden (or not) of LOGGING ON/USE – Impact on normal daily routine/choices<br>▪ FIT OF iSUPPORT with values and culture
**10. Do you think iSupport is/was particularly useful in COVID times? Why?**<br>**10.1.** Do you think iSupport 'makes more sense' now that we are dealing with a pandemic? Do you think it can help support carers if we are ever again in a similar situation? Why?	▪ COVID & NEED/RELEVANCE OF iSUPPORT

USABILITY AND EXPERIENCE OF USE	
**11. What was your overall impression of using iSupport?**<br>**12. What were the most positive aspects/advantages?**<br>**13. If any, which were the most negative/challenging aspects?**<br>**13.1.** Did you have any issues with technology?<br>**13.2.** Did you need to contact the e-coach? How often did you have contact with the e-coach?<br><br>**14. Was iSupport easy to access? (including 'read aloud function')**<br>**14.1.** Did you have any problems logging on?<br>**14.2.** Do you think most carers would have been able to follow the logging on steps?<br>**15. Was iSupport easy to use?**<br>**15.1.** To what extent do you consider that most carers would be able to use the program without help?<br>**15.2.** Did you find any error or problem when using the program? If so, how did you resolve it?	▪ iSUPPORT – OVERALL IMPRESSION<br>▪ iSUPPORT – POSITIVES<br>▪ iSUPPORT - NEGATIVES<br><br><br><br><br>▪ ISSUES AROUND ACCESS/LOGGING ON<br>▪ EASE OF USE (Once logged on)
**16. What do you think about the 'look' (visual appearance) of iSupport?**<br>**16.1.** Was it appealing to look at?<br>**16.2.** What do you think about the design and images used? Did you find they fit with your value system and cultural background?<br>**16.3.** What do you think about how the text was organized?<br>**17. What is your opinion about the language used?**<br>**17.1.** To what extent do you consider the language to be plain and accessible to most carers?<br>**17.2.** Did you find any negative, offensive, or inadequate term or expression? Did the language used fit with your value system and cultural background?	▪ VISUAL APPEAREANCE (issues around)<br>▪ LANGUAGE (issues around)
**18. What are your thoughts about the content of the modules?**<br>**18.1.** What do you think about the usefulness or relevance of the modules?<br>**18.2.** Was there any kind of information that you did not find helpful? Can you think of an example?<br>**18.3.** Was there any kind of information that was missing? Can you think of an example?<br>**18.4.** Was there any information that you found unnecessary or inadequate?	▪ CONTENT OF iSUPPORT<br>▪ WHAT IS MISSING (regarding iSupport content)?
MECHANISMS OF CHANGE	
**19. What impact has iSupport had, if any, in your health and wellbeing?**<br>**20. What is it about iSupport that has contributed to these changes/impact?**<br>**20.1** What aspects of iSupport, if any, do you think have helped the most in making a difference to the way you deal with your caring role?<br>**21. What do you feel was the most significant change in the way you deal with your caring role after accessing iSupport?**<br>**21.1.** Have you learnt more about how to deal with your caring role? In what way?<br>**21.2.** Have you implemented anything you learnt in iSupport into your day-to-day activities? Can you think of an example?<br>**21.3.** In what way, if any has iSupport impacted the person you are caring for and your relationship with him/her?<br>**22. Would you recommend iSupport to other carers? Why?**<br>**22.1.** Which carers do you think would benefit most from using iSupport?	▪ IMPACT (of iSupport) ON HEALTH/WELLBEING<br>▪ What is it about iSUPPORT that generates (doesn't) impact?<br>▪ IMPACT ON CARING ROLE – Learning new things<br>▪ IMPACT ON CARING ROLE – Doing things differently<br>▪ IMPACT ON PLWD<br>▪ IMPACT ON Relationship PLWD/CARER<br>▪ RECOMMENDING iSupport (target audience)
