# Supplementary material for: A Process Evaluation of the UK Randomised Trial Evaluating ‘iSupport’, an Online e-Health Intervention for Adult Carers of People Living with Dementia
Source: Behav Sci (Basel). 2025 Aug 15;15(8):1107. doi: 10.3390/bs15081107 (PMC12382822; doi:10.3390/bs15081107)
Supplement: Supplementary file 1 [file behavsci-15-01107-s001.zip › Supplementary File S5.pdf]

OBJECTIVE 1: To examine the usability and the acceptability of iSupport

THEME 1: The process of ‘navigating iSupport’

1.1 Complex flow
1.2 Too many ‘clicks’

THEME 2: Factors driving the tension between provision of iSupport via Hardcopy vs Online

2.1 IT literacy
2.2 Personal preference

THEME 3: The way ‘it looks’ – clear but wordy

3.1 Clear and simple
3.2 Less text – too wordy

OBJECTIVE 2: To determine participant level of engagement and adherence to iSupport

THEME 4: Do I have time? Do I want to make time for iSupport?

4.1 Something else I had to do
4.2 Prioritizing

THEME 5: Choosing how and when to engage with iSupport

5.1 Dipping in and out
5.2 Module order
5.3 The role of ‘reminders’

OBJECTIVE 3: To examine the contextual factors influencing the uptake and implementation of iSupport

THEME 6: The role of motivations and expectations

6.1 My responsibility as a carer
6.2 Curiosity

THEME 7: iSupport content – training vs information

7.1 Just information vs ‘reflexive’ approach
7.2 Being trained/tested

THEME 8: Too complex? Too simple - balance

8.1 One-stop shop for dementia
8.2 Too basic

THEME 9: The timing – who is iSupport pitched at?

9.1 Caring/Illness stages – the right time
9.2 Good ‘starting point’

THEME 10: iSupport on its own is not good enough

10.1 Need for a ‘human element’
10.2 Interaction and feedback are missing

OBJECTIVE 4: To examine the extent to which iSupport may have changed behaviours beyond the intervention

THEME 11: Improved wellbeing of carers

11.1 I feel reassured
11.2 It’s a two-way thing
11.3 Acknowledged, less alone

THEME 12: Improved communication and relations with PLWD

12.1 Awareness about feelings and opinions of PLWD
12.2 Able to ‘go with the flow’
12.3 Better informed

THEME 13: iSupport has made no difference

13.1 Content – nothing earth-shattering
13.2 Nothing I could gain for it (after all these years)
