# Supplementary material for: A Process Evaluation of the UK Randomised Trial Evaluating ‘iSupport’, an Online e-Health Intervention for Adult Carers of People Living with Dementia
Source: Behav Sci (Basel). 2025 Aug 15;15(8):1107. doi: 10.3390/bs15081107 (PMC12382822; doi:10.3390/bs15081107)
Supplement: Supplementary file 1 [file behavsci-15-01107-s001.zip › Supplementary File S6.pdf]

SUPPLEMENTARY FILE S6 - ADDITIONAL TABLES

Table S1. iSupport usage data for process evaluation participants (interview participants and online survey participants) and trial intervention group participants.

	**Online Survey Part. (N=93)**	**Interview Part. (N=52)**	**Intervention group (N=175)**
**Median [IQR]**	79 [36, 45]	79 [50, 149]	49 [5, 104]
	**N (%)**	**N (%)**	**N (%)**
**Time on iSupport (min.)**			
0	4 (4.3)	4 (7.7)	32 (18.3)
<30min	16 (17.2)	6 (11.5)	**39 (22.3)**
30min-1hr	17 (18.3)	6 (11.5)	30 (17.1)
> 1hr ≤ 1.5hr	**18 (19.4)**	**18 (34.6)**	25 (14.3)
> 1.5hr ≤ 2hr	10 (10.8)	2 (3.8)	14 (8)
> 2hr ≤ 3hr	12 (12.9)	6 (11.5)	15 (8.6)
> 3hr ≤ 4hr	6 (6.5)	2 (3.8)	8 (4.6)
> 4hr ≤ 5hr	4 (4.3)	4 (7.7)	5 (2.9)
> 5hr ≤ 6hr	3 (3.2)	2 (3.8)	4 (2.3)
> 6hr ≤ 7hr	2 (2.2)	1 (1.9)	2 (1.1)
> 7hr ≤ 7.5hr	1 (1.1)	1 (1.9)	1 (0.6)

Table S2 Online survey responses (non SUS items) of those participants who spent a minimum of 30 minutes on iSupport (n=73).

	Not important	2	3	4	Extremely important
**Think about any challenges that you might have encountered when using iSupport and rate their importance**	**n(%)**	**n(%)**	**n(%)**	**n(%)**	**n(%)**
Amount of time needed to use iSupport	15 (20.5)	11 (15.1)	13 (17.8)	**17 (23.3)**	**17 (23.3)**
Use and complexity of iSupport platform	11 (15.1)	12 (16.4)	**21 (28.8)**	17 (23.3)	12 (16.4)
Lack of confidence/experience in using technology	**27 (37)**	17 (23.3)	15 (20.5)	11 (15.1)	3 (4.1)
**Think about advantages in using iSupport and rate their importance**					
Autonomy and self-paced programme	2 (2.7)	2 (2.7)	8 (11)	27 (37)	**34 (46.6)**
Access to diverse sources of information	4 (5.5)	5 (6.8)	18 (24.7)	**23 (31.5)**	**23 (31.5)**
Ability to choose the most relevant modules/sessions/activities	3 (4.10	3 (4.1)	11 (15.1)	19 (39.7)	**27 (37)**
Ability to use iSupport from my own home	2 (2.7)	1 (1.4)	5 (6.8)	20 (27.4)	**45 (61.6)**

Table S3 Online survey responses (non SUS items) of those participants who spent a minimum of 30 minutes on iSupport (n=73).

	Strongly disagree	Disagree	Neither agree nor disagree	Agree	Strongly agree
	**n(%)**	**n(%)**	**n(%)**	**n(%)**	**n(%)**
I have the right resources to be able to use iSupport	2 (2.7)	1 (1.4)	2 (2.7)	31 (42.5)	**37 (50.7)**
The language used in iSupport was easy to understand	0 (0)	1 (1.4)	7 (9.6)	**44 (60.3)**	21 (28.8)
I consider that most carers would be able to use iSupport without help	**4 (5.5)**	**21 (28.8)**	20 (27.4)	**26 (35.6)**	2 (2.7)
I was able to use iSupport without any help	0 (0)	6 (8.2)	9 (12.3)	**33 (45.2)**	25 (34.2)
I liked using iSupport to learn about dementia	5 (6.8)	15 (20.5)	15 (20.5)	20 (27.4)	**18 (24.7)**
I liked using iSupport to learn about my role as a carer	6 (8.2)	13 (17.8)	15 (20.5)	**22 (30.1)**	17 (23.3)
iSupport has taught me something about dementia	4 (5.5)	8 (11)	13 (17.8)	**34 (46.6)**	14 (19.2)
iSupport has taught me something about my caring role	5 (6.8)	6 (8.2)	12 (16.4)	**34 (46.9)**	16 (21.9)
I can easily apply the content of iSupport to my daily role as a carer	4 (5.5)	7 (9.6)	18 (24.7)	**33 (45.2)**	11 (15.1)
